# Mixing Approaches in Enhancing the Capacitive Performance of rGO-Based Hybrid Electrodes

**DOI:** 10.3390/ma18112460

**Published:** 2025-05-24

**Authors:** Svetlana Veleva, Delyana Marinova, Sonya Harizanova, Violeta Koleva, Elefteria Lefterova, Maria Shipochka, Ognian Dimitrov, Antonia Stoyanova, Radostina Stoyanova

**Affiliations:** 1 Institute of Electrochemistry and Energy Systems, Bulgarian Academy of Sciences, 1113 Sofia, Bulgaria; svetlana_veleva@iees.bas.bg (S.V.); edl@iees.bas.bg (E.L.); ognian.dimitrov@iees.bas.bg (O.D.); antonia.stoyanova@iees.bas.bg (A.S.); 2 Institute of General and Inorganic Chemistry, Bulgarian Academy of Sciences, 1113 Sofia, Bulgaria; manasieva@svr.igic.bas.bg (D.M.); sonya@svr.igic.bas.bg (S.H.); vkoleva@svr.igic.bas.bg (V.K.); shipochka@svr.igic.bas.bg (M.S.)

**Keywords:** hybrid composites, nickel manganese oxides, reduced graphene oxide, hybrid supercapacitors

## Abstract

Combining carbon materials with oxides in a hybrid electrode is an effective way to control supercapacitor performance in terms of balancing energy and power density with cycling stability. However, it is still unclear how the mixing method of each component affects the supercapacitor performance. In this study, the influence of mixing reduced graphene oxide (rGO) with ilmenite-type nickel-manganese oxide (NiMnO_3_) on the capacitive behaviour of the resulting composites is investigated. Two preparation methods are compared: mechanical mixing and ultrasonication. The capacitive characteristics were evaluated in hybrid supercapacitors using 6M KOH electrolyte. The bulk, surface, and morphological changes of the composites after long-term cycling were probed by EIS and ex situ XRD, XPS, and SEM analyses. It is established that the composites obtained by mechanical mixing exhibit better performance due to the stable contact between rGO and NiMnO_3_ particles, favourable surface reactions with KOH and preserved morphology of rGO. These findings indicate that efficient hybrid electrodes can be achieved without relying on costly synthesis techniques such as hydrothermal or ultrasonic treatments.

## 1. Introduction

Today, supercapacitors have attracted both research and application interest due to their specific properties such as high power density and excellent cycle stability, combined with relatively low cost and environmentally friendly characteristics [1,2,3]. This is a consequence of their unique ability to store energy [1,2,3]. There are two main mechanisms of energy storage: one is the electrical double-layer reaction that takes place at the electrode–electrolyte interface, and the other is the Faradaic process that is expressed in rapid surface redox reactions with participation of the electrode materials and electrolyte ions [3,4]. The main class of electrodes that operate by double layer electrochemical reactions are carbonaceous materials, while oxides and hydroxides underlie the Faradaic reactions [1,2,3,4]. The carbonaceous electrodes demonstrate superior cycling stability, but the power energy is still unsatisfactory [1,2,3,4]. Unlike carbon electrodes, oxide/hydroxide electrodes deliver higher energy and power density, but their low electronic conductivity prevents them from achieving optimum characteristics [5]. In this respect, the combination of carbonaceous materials with oxide/hydroxides in a hybrid electrode is an effective way to control the supercapacitor performance in terms of balancing the energy and power density with cycling stability [6].

Recently, graphene has emerged as a competitive electrode for supercapacitor applications due to its unique electrical conductivity and flexibility [7]. Compared to graphene, reduced graphene oxide (rGO) has recently been the subject of intense research because it is cheaper and easier to synthesize [8,9]. Among the oxides and hydroxides, the best supercapacitor performance has been found for mixed nickel-manganese oxides with an ilmenite-type structure, NiMnO_3_ [10,11]. The performance of rGO and NiMnO_3_ can further be improved by the formation of composites between them [12,13,14,15]. The improved supercapacitor performance is associated with a high capacitance of NiMnO_3_ and the good conductivity of rGO [12,13,14,15]. The morphology of the composites also has an impact on their supercapacitor properties: for the Cu_2_O/rGO composite, the highest capacitance is achieved when Cu_2_O adopts an octahedral particle shape [16]. The rGO component has also been used as a multifunctional conductive binder, resulting in improved supercapacitor performance [17]. All composites have been obtained by a special developed procedure including the hydrothermal route [12,13,14,15,16] and electrostatic co-precipitation [18]. Furthermore, to improve the contact between oxides and rGO components, the hydrophilic H-C_3_N_4_ is additionally used as a “bridge” between them [19]. The capacitive performance of the composites also depends on the choice of electrolyte: the alkaline electrolytes with high ionic conductivity give the highest capacitance and energy density compared to Na_2_SO_4_/H_2_SO_4_ electrolytes [20]. Despite these studies, questions remain as to how the way in which the individual components are mixed affects the properties of the supercapacitor, and how stable the composites are during long-term supercapacitor cycling.

Herein, we explore the concept of forming hybrid composites between electrically conductive rGO and high-power oxides with the aim of monitoring the effect of the mixing on the capacitive performance of the composites. Ilmenite-type nickel-manganese oxides are selected as oxide electrodes because they provide a good balance between capacitive performance and environmental concerns [10,21,22]. Additionally, NiMnO_3_ exhibits enhanced redox activity, electron-ion conductivity, and structural stability compared to pure MnO_2_ and NiO due to the synergy between Ni and Mn in the crystal structure [23,24]. For the preparation of NiMnO_3_/rGO composites, two simple and fast synthetic procedures are adopted, namely mechanical mixing and ultrasonic treatment of the components. Capacitive performance of the composites is evaluated in hybrid supercapacitor devices with a 6M KOH electrolyte. In comparison with LiOH and NaOH, KOH has higher ionic conductivity and better ionic mobility and offers higher electrochemical stability over repeated charge/discharge cycles [25]. The bulk, surface and morphological changes of the composites at the end of the supercapacitor life are probed by ex situ XRD, XPS, and SEM analyses. The combination of all these techniques enables the identification of new relationships between the manner of mixing in the composites, the surface reactivity of the functional groups, and capacitance performance.

## 2. Materials and Methods

This section describes the materials used to prepare the composites, the preparation methods, the structural and morphological characterization techniques applied, and the electrochemical methods used to evaluate the performance of the prepared electrodes.

### 2.1. Composites Preparation

Nickel-manganese oxide, Ni_1.5_Mn_1.5_O_3_, with an ilmenite type structure is obtained by thermal decomposition of precipitated Ni_0.5_Mn_0.5_CO_3_ at 400 °C. The details are given elsewhere [10]. For the simplicity, the ilmenite phase Ni_1.5_Mn_1.5_O_3_ will be denoted with NMO. The reduced graphene oxide is a commercial product that was kindly provided by the company Graphit Kropfmühl GmbH (Hauzenberg, Germany).

The composites between rGO and NMO (1:9 weight ratio) were prepared by homogenization using two different mixing methods. According to the first one, rGO was mixed with NMO using a planetary centrifugal mixer ARE-250 CE (THINKY, Tokyo, Japan) for 10 min at a speed of 500 rpm, and then for the next 50 min at 2000 rpm. The second method involves ultrasonic mixing (the conditions being power 60 W for two and a half hours), preceded by preliminary mixing in an agate mortar. Both methods used small amounts of ethanol during mixing. For the sake of convenience, the composites obtained by mechanical (M) and ultrasonic (U) mixing will be denoted as NMO/rGO-M and NMO/rGO-U, respectively. It worth mentioning that the ratio between rGO and NMO (1:9 weight ratio) is quite comparable to previously published data on rGO composites in supercapacitors [26].

### 2.2. Structural and Morphological Characterization of Composites

The oxides and composites were characterized with X-ray powder diffraction (XRD) using a Bruker Advance D8 diffractometer (Bruker, Billerica, MA, USA) with a LynxEye detector (CuKα radiation) (Bruker, Billerica, MA, USA). The specific surface area and pore texture of the composites were evaluated by low-temperature nitrogen adsorption isotherms using the Quantachrome NOVA 1200e instrument (AntonPaar Quanta Tech Inc., Boynton Beach, FL, USA). The pore size distribution curves were calculated with the Barett–Joyner–Halenda method. The morphology of composites and electrodes was monitored with scanning electron microscopy (SEM) (JEOL JSM 6390 microscope, Tokyo, Japan). The surfaces of electrodes were examined with X-ray photoelectron spectroscopy (XPS) analysis (VG Escalab II system with Al Kα radiation, Thermo Fisher Scientific, Waltham, MA, USA).

### 2.3. Electrochemical Characterization of Composites

Electrochemical characterization of composites includes the evaluation of key performance parameters such as specific capacitance, energy/power density, and cycling stability. Two-electrode cells were used to characterize the electrochemical performance of the electrodes. The cell consists of a positive electrode composed of 80 wt.% NMO/rGO and a negative electrode containing 80 wt.% activated carbon (YP-50F, “Kuraray Europe” GmbH, Main, Germany). Polyvinylidene difluoride (PVDF, 10 wt.%, Merck, St. Louis, MO, USA) and graphite ABG 1005 EG1 (10 wt.%, Superior Graphite, Chicago, Illinois, USA) were used as binder and conductive additive, respectively. The mass ratio of the two electrodes was maintained close to 1:1. The mass of the electrodes was approximately 0.003 g, with the surface area being 0.636 cm^2^. The active material was deposited on Ni foam as a current collector, the electrode surface being approximately 0.8 cm^2^. The prepared electrodes were dried at 80 °C for 6 h and pressed under a pressure of 20 MPa. The electrode package was installed in an electrochemical cell with a Viledon separator and a 6 M KOH electrolyte solution.

Cyclic voltammetry (CV) experiments were performed using a Multi PalmSens system (model 4, 3991 CL Houten, The Netherlands) in a voltage window from 0.05 to 1.2 V and varying scan rates of 1, 10, 20, and 50 mVs^−1^. The specific capacitance Cs (Fg^−1^) was determined based on cyclic voltammetric curves using the following equation:

(1) $$C_{s}=\frac{4I}{m\frac{dV}{dt}}$$

where I is the current (A), dV/dt is the voltage scan rate (Vs^−1^), and m is the total mass of the active materials in both electrodes (g). The number 4 is needed to report the capacitance for a single electrode [27].

Charge–discharge curves were recorded on an Arbin Instrument System BT-2000 (Arbin Instruments, College Station, TX, USA). Capacitance (Fg^−1^) was calculated from the charge–discharge curves using the following equation:

(2) $$C=\;\frac{I\Delta t}{mdV}$$

where I (A), ∆t (s), m (g), and ∆V (V) are the discharge current, discharge time, mass of the active material, and voltage window, respectively.

Based on the capacitance calculated using Equation (2), the energy densities (E, Whkg^−1^) and power densities (P, Wkg^−1^) were subsequently determined according to Equations (3) and (4), respectively.

(3) $$E=\frac{C\Delta V^{2}}{7.2}$$

and

(4) $$P=\frac{3600E}{t}$$

Additionally, electrochemical impedance spectroscopy (EIS) measurements were conducted on the same Multi PalmSens system (model 4, 3991 CL Houten, The Netherlands), covering a frequency range from 10 MHz to 1 mHz.

## 3. Results and Discussion

### 3.1. Structure and Morphology of NMO/rGO Composites

**Figure 1:**
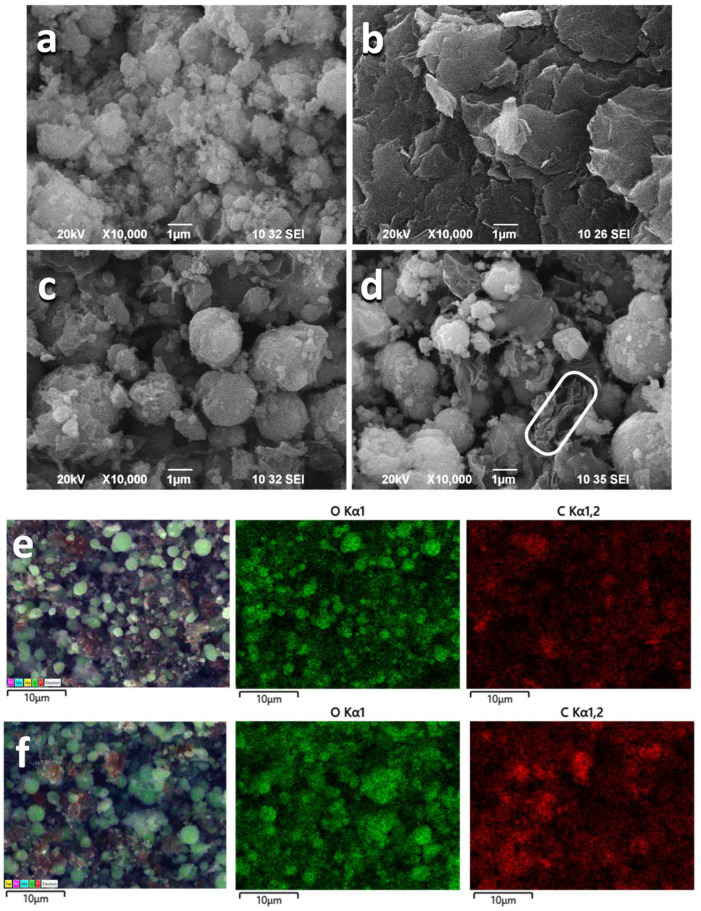
SEM images of NMO (**a**), rGO (**b**), NMO/rGO-M (**c**), and NMO/rGO-U (**d**). The separate flake-like rGO particles are indicated in the frame rGO is indicated on NMO/rGO-U (**d**). C and O element mapping for NMO/rGO-M (**e**) and NMO/rGO-U (**f**).

The mechanical and ultrasonic homogenization of rGO and NMO yield composites, in which each individual component retains its own structure [10,28] (Figure S1). The morphology of the individual NMO and rGO components is quite different (Figure 1): NMO displays micron-sized aggregates that are predominantly spherical, whereas the morphology of rGO consists of flake-like micrometric particles measuring about 1–5 µm in length and below 0.1 µm in thickness. After mixing NMO with rGO, the morphology of the composite undergoes changes, manifested by the formation of spherical aggregates with a more homogeneous diameter distribution around 2–6 μm (Figure 1). In addition, the flake-like particles are still visible for the composites. This means that after mechanical and ultrasonic treatment, rGO has a dual role: firstly, it breaks down the NMO aggregates, and then it acts as an adhesive to bind the NMO particles into spherical aggregates. However, this is not a chemical interaction (see the XPS data below). The comparison of the morphologies of NMO/rGO-M and NMO/rGO-U evidences that a more homogeneous distribution of the spherical aggregates and a lower level of separate flake-like rGO particles is achieved when mechanical mixing is used. To support this suggestion, Figure 1 gives the C and O element mapping of the composites. As one can see, the C and O distribution is more homogeneous for NMO/rGO-M than that for NMO/rGO-U. This indicates that the manner in which rGO and NMO are mixed depends on whether mechanical or ultrasonic treatment is applied.

**Figure 2:**
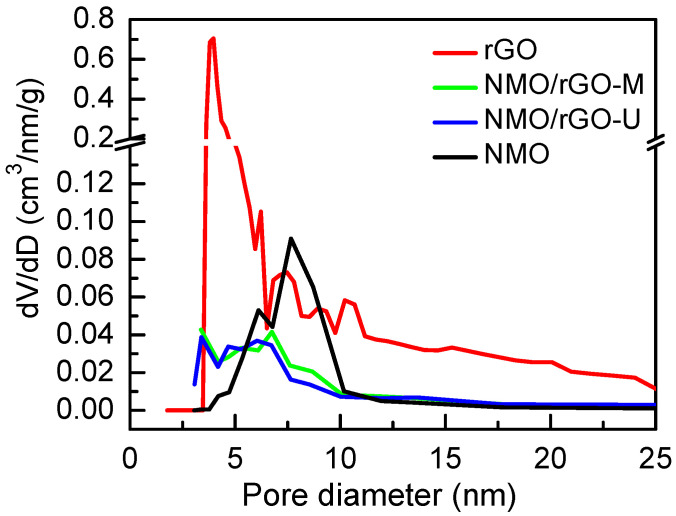
Pore size distribution curves of NMO, NMO/rGO-M, NMO/rGO-U, and rGO.

The further effect of rGO on the composite formation is monitored by the nitrogen adsorption curves (Figure S2). The specific surface area and total pore volume of NMO are lower than those of rGO (Table S1). In NMO, the pores are mainly distributed between 4 and 10 nm (more than 70%), whereas rGO has a large proportion of pores in the 3–5 nm range (more than 85%). The composites bring the texture properties that are typical of the individual NMO and rGO components (Figure 2 and Figure S2). The specific area of NMO/rGO-M and NMO/rGO-U is close to that of the main component NMO (Table S1). At first glance, the pore size distribution curves for the composites appear to be the sum of the corresponding curves for the individual components, resulting in pores between 3 and 10 nm dominating the composite texture. However, closer inspection reveals a trend towards a slight decrease in specific surface area when switching from ultrasonic to mechanical mixing (Table S1). In the same order, the total pore volume decreases too (Table S1). These data suggest that some of the NMO pores are blocked by rGO during the composite formation. Comparison of the pore size distribution curves implies that the blocked pores are mainly between 7–8 nm. In addition, the pore blockage is more pronounced in the mechanical treatment than in the ultrasonic treatment. This reveals once again that the way of mixing is of significance for the preparation of NMO/rGO composites.

### 3.2. Supercapacitor Performance

**Figure 3:**
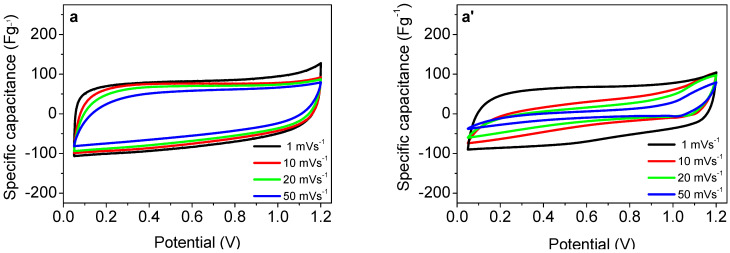

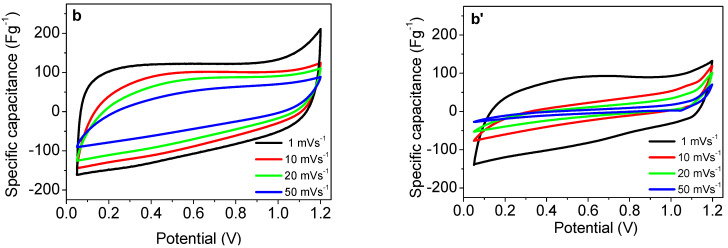
CV plots of NMO/rGO-M (**a**,**a′**) and NMO/rGO-U (**b**,**b′**) at different scan rates from 1 to 50 mVs^−1^ before and after GCD test.

Figure 3 compares the CV curves of NMO/rGO-M and NMO/rGO-U composites, while the CV plots of rGO at 1 mVs^−1^ and 50 mVs^−1^, recorded before and after the GCD test, are presented in Figure S3. The NMO/rGO-M displays a rectangular CV curve with no redox waves. The calculated capacitance from the CV curves appears to be insensitive to the applied scan rates in the range of 1–50 mVs^−1^. On the one hand, these data indicate that the NMO/rGO-M functions in the electrochemical cell as a result of the capacitive reactions. On the other hand, the stable CV curve shapes of NMO/rGO-M at different scan rates indicate a good electrode integrity and ion accessibility. In contrast to NMO/rGO-M, the CV curves of NMO/rGO-U (as well as their shapes) are more dependent on the scan rate, implying an additional contribution of Faradaic-like properties to the capacitive ones. This leads to a higher capacitance of NMO/rGO-U than that of NMO/rGO-M, especially at lower scan rates. At fast scan rates, where the capacitance reactions dominate, the capacitance of NMO/rGO-M is slightly higher than that of NMO/rGO-U. This provides evidence that the manner in which rGO and NMO are mixed affects the capacitance properties: the more homogeneous the distribution, the more stable the capacitance over a wide range of scan rates. The stability of capacitive characteristics of NMO/rGO-M is consistent with the SEM data, where a more homogeneous distribution between rGO and NMO is observed after the mechanical mixing.

**Figure 4:**
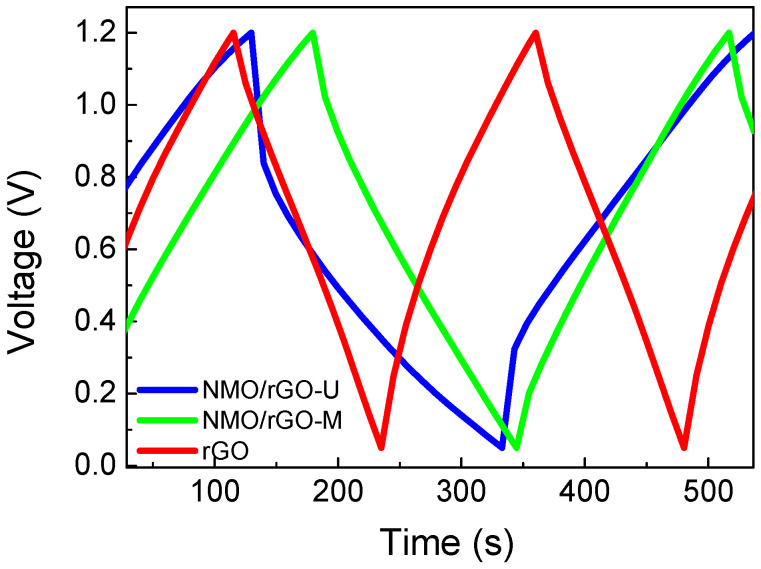
Galvanostatic charge/discharge curves of NMO/rGO-M, NMO/rGO-U, and rGO under current load of 240 mAg^−1^.

**Figure 5:**
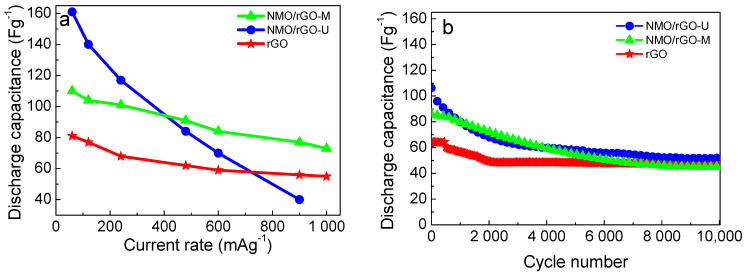
Comparison of discharge capacitance as function of current load for SCs with compositions of NMO/rGO-M, NMO/rGO-U, and rGO (**a**); long-term cyclic stability under current load of 240 mAg^−1^ (**b**).

To evaluate the capacitance performance of NMO/rGO-M and NMO/rGO-U, the galvanostatic test is performed (Figure 4 and Figure 5). As in the case of the CV curves, the galvanostatic curves display typical shapes for the capacitive reactions (Figure 4). At low current load, the capacitance of NMO/rGO-U is higher than that of NMO/rGO-U, but the capacitance decreases rapidly as the current load increases (Figure 5a). This behaviour is probably due to the weaker contact between rGO and NiMnO_3_ particles in the ultrasonically treated sample, which limits the effective charge transfer at higher current values. As a result, the capacitance characteristics of NMO/rGO-U become worse than those of NMO/rGO-M at high current loads. It is worth noting that the NMO/rGO-M composite outperforms (in terms of capacitance and rate capability) the rGO, which is known to exhibit a good capacitance performance [29]. The better capacitance properties of NMO/rGO-M are due to a more homogeneous mixing of the individual components compared to NMO/rGO-U.

The capacitive properties of NMO/rGO-U and NMO/rGO-M are further differentiated by testing the long-term cycling stability at 240 mAg^−1^ for 10,000 cycles (Figure 5b). This duration allows for a reasonable assessment of electrode degradation and capacitance retention under prolonged use. At the beginning, the capacitance increases in the order rGO < NMO/rGO-M < NMO/rGO-U, but after 10,000 cycles, the capacitance tends to be close for all samples. This means that rGO (75%) and NMO/rGO-M (55%) achieve better cycling stability, and NMO/rGO-U (45%) worse. The improved cycling stability of NMO/rGO-M and rGO is associated with a lower IR drop in comparison with that for NMO/rGO-U: IR is 0.13 V, 0.18 mV and 0.36 V for rGO, NMO/rGO-M, and NMO/rGO-U, respectively (Figure 4). The cycling stability of NMO/rGO-M is comparable with the best one reported previously for the composite Sr_2_Ni_2_O_5_@15%rGO: capacity retention of 68.5% after 10,000 cycles [30]. Although, after 10,000 cycles, a decrease in specific capacitance is observed, the shape of the CV curve remains almost unchanged for both NMO/rGO-U and NMO/rGO-M (Figure 3b,b′), indicating that the charge storage mechanism is preserved. Therefore, the observed performance loss is likely related to partial structural/morphological degradation and/or reduced conductivity, rather than a fundamental change in the electrochemical behaviour [31].

**Figure 6:**
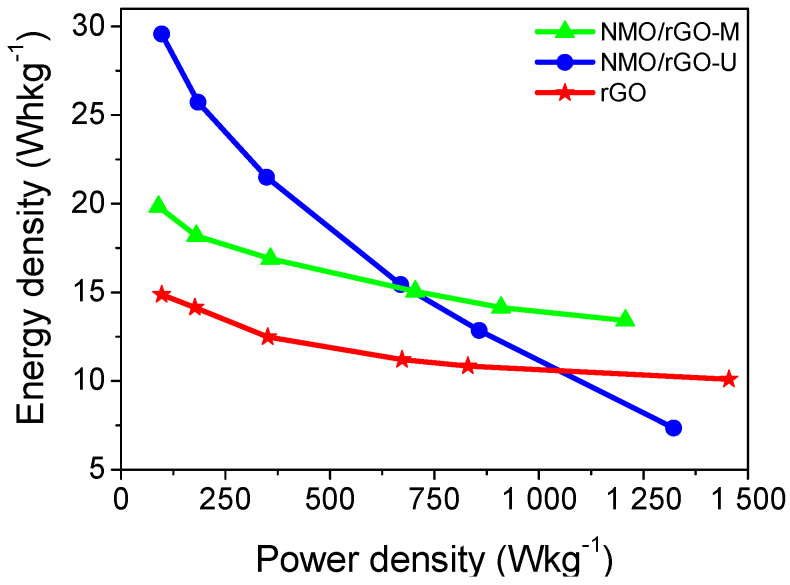
Ragone plot for SCs with NMO/rGO-M, NMO/rGO-U, and rGO.

Another parameter used for the evaluation of the supercapacitor performance of the composites is the relationship between energy density and power density (Figure 6). Using the Ragone plot, higher energy density at high power is observed for the NMO/rGO-M composite: energy density of 15 Whkg^−1^ at power of 1200 Wkg^−1^. Importantly, the energy density of NMO/rGO-M is higher than that of the individual rGO component irrespective of the power density. Compared to NMO/rGO-M, the NMO/rGO-U composite has high energy density only at low power density, which is unacceptable for supercapacitor applications. From these data, it can be inferred that the NMO/rGO-M composite outperforms both NMO/rGO-U and rGO. In addition, the energy density and power density of NMO/rGO-M exceed those of previously reported composites: the hydrothermally prepared NiMnO_3_/rGO composite exhibits 8.4 Whkg^−1^ energy density at a power density of 764 Wkg^−1^ [13]. It should be noted that the comparison should be taken with caution as we used an alkaline electrolyte, whereas Na_2_SO_4_ has been used previously [13]. Moreover, the performance of NMO/rGO-M obtained by a simple mechanical mixing process is comparable to that of composites prepared by complex hydrothermal methods: in 3 M KOH electrolyte, Mn-Co oxide@/rGO composites have an energy density of 55.55 Whkg^−1^ at a power density of 972.73 Wkg^−1^ [32], while for ZnMn_2_O_4_/rGO, an energy density of 40.2 Whkg^−1^ at a power density of 1125 Wkg^−1^ is reported [33].

### 3.3. Ex Situ Characterization

To understand the long-term supercapacitor performance of the composites, the CV curves of the electrodes after 10,000 cycles are shown in Figure 3. For all the composites, the CV curves retain their rectangular shape at low scan rates, while the fast scan rates cause changes in the curve shape and the magnitude of the capacitance. The decrease in the specific capacitance with increased scan rates can be related to an increase in the electrode resistance, as well as with a decrease in the electrode accessibility by the electrolyte. The comparison shows that these changes are more significant for the composites obtained by ultrasonic mixing. This is an indication that the composites undergo some changes after prolonged cycling, especially NMO/rGO-U.

**Figure 7:**
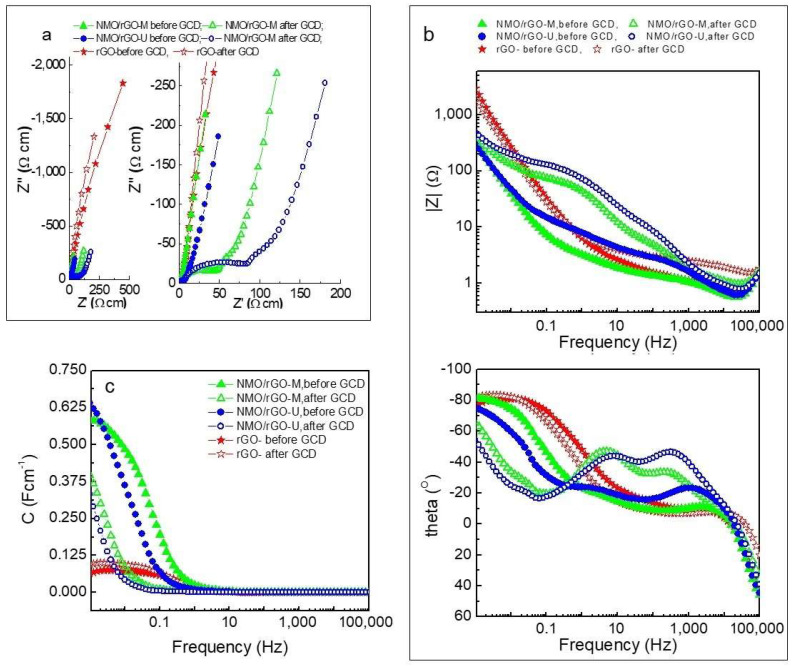
EIS of studied NMO/rGO-M and NMO/rGO-U composites before and after 10,000 cycles: (**a**) Nyquist plots full range (**left**) and zoomed (**right**), (**b**) Bode plot—IZI vs. frequency (**up**) and Theta vs. frequency (**down**); (**c**) specific capacitance as a function of frequency.

EIS was conducted to gain deeper insight into the electrical properties of the electrodes, electrolyte resistance, and other charge transfer processes in the supercapacitors (Figure 7). It is evident that after cycling, a new large semicircle appears in the Nyquist plots (Figure 7a) for both the NMO/rGO-M and NMO/rGO-U electrodes. This semicircle corresponds to high resistance Rph (35 Ωcm for NMO/rGO-M and 72 Ωcm for NMO/rGO-U), leading to a significant increase in the overall resistance RΣ (61.4 Ωcm for NMO/rGO-M and 108.2 Ωcm for NMO/rGO-U). The reasons for these changes are likely related to structural, chemical, or morphological transformations, such as formation of a new phase, degradation of the contact between individual components (high resistance at the interfacial boundaries), growth of crystallites, and other factors. As a result, a new process with distinct time constant is clearly observed in the Bode plots at lower frequencies (Figure 7b). This is reflected as a second peak in the theta vs. frequency plot and a second corresponding step in the ∣Z∣ vs. frequency plot.

Two new processes with distinct time constants are clearly observed in the Bode plots. This is reflected as two peaks in the Θ vs. frequency plot (Figure 7c) and two corresponding steps in the ∣Z∣ vs. frequency plot (Figure 7c). Ultrasonic treatment appears to accelerate these structural and electrochemical modifications, leading to an increase in resistance and a slight decrease in the capacitance of the electrode (Figure 7c).

The structural and morphological stability after electrode cycling are also examined by ex situ XRD and SEM. Figure S4 shows the ex situ XRD patterns of the electrodes after 10,000 cycles. In general, the XRD peaks of the ilmenite phase in the NMO/rGO-M and NMO/rGO-U electrodes remain unchanged, indicating their chemical and structure stability during the prolonged cycling in the KOH electrolyte (Figure S4). However, the structural stability of the composite electrodes cannot be used to explain the increase in the resistance Rph.

**Figure 8:**
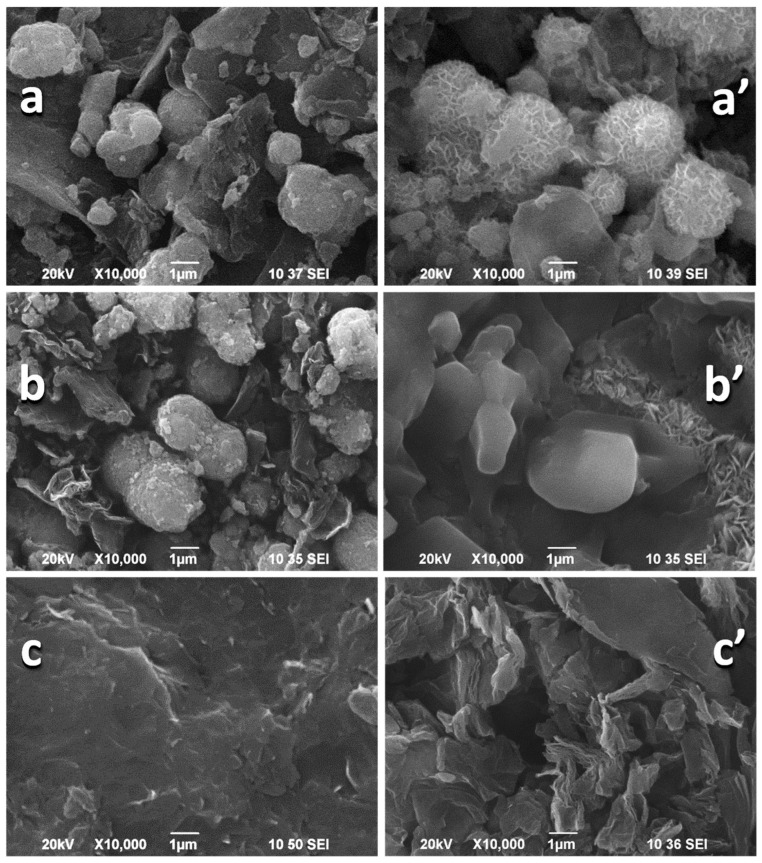
SEM images of pristine electrodes (**left**) and electrodes after 10,000 cycles (**right**): NMO/rGO-M (**a**,**a′**), NMO/rGO-U (**b**,**b′**), and rGO (**c**,**c′**). The crystal needles seen on the image (**b′**) correspond to crystallized KOH from the electrolyte.

A further comparison of the morphology of the pristine and cycled electrodes is shown in Figure 8. As can be seen, the spherical morphology of NMO is maintained after electrode fabrication and the contact between the spherical aggregates is ensured by the rGO component. After the electrochemical test for 10,000 cycles, there is a change in the morphology of the electrodes, depending on the method of the composite formation. For NMO/rGO-M, the surface of the spherical aggregates becomes frizzled. However, there is still contact between the aggregates. For NMO/rGO-U, it appears that the spherical aggregates composed of NMO are detached from the rGO. The loss of contact between NMO and rGO is in good agreement with the increase in resistance Rph. Compared to the composite electrodes, the rGO electrode undergoes more severe morphological changes: after 10,000 cycles, the flat particles are broken and wrinkled. This is a SEM evidence that the rGO component in the composites is mainly attacked by the electrolyte composed of 6M KOH. It is worth mentioning that the capacitive properties of rGO depends strongly on the electrolyte composition: it has been shown that the more active species in aqueous solutions are K^+^ and OH^−^ ions compared to Na^+^, SO_4_^2−^, Cl^−^ [34]. Returning to the sample studied by us, it can be inferred that the morphological changes of rGO are responsible for the observed detachment of rGO from the NiMnO_3_ particles after prolonged cycling.

**Figure 9:**
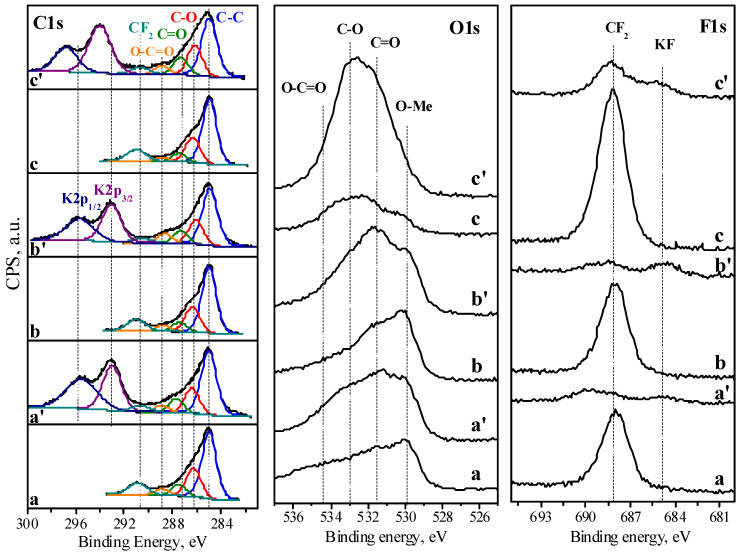
XPS spectra in the C1s, O1s, and F1s binding energy ranges for pristine electrodes and electrodes after 10,000 cycles: NMO/rGO-M (**a**,**a**′), NMO/rGO-U (**b**,**b**′), and rGO (**c**,**c**′).

To track surface changes in the electrodes, ex situ XPS analysis in the ranges of C1s, O1s, and F1s binding energies is carried out (Figure 9). The C1s XPS spectra of all samples display an intensive signal at 284.8 eV, due to sp^2^-hybridized graphitic carbon. Superimposed on the main signal, additional low-intensity signals at 286.3 eV, 287.8 eV, and 288.8 eV can also be deconvoluted. The additional peaks can be attributed to carbon atoms in hydroxyl/epoxide groups (C-O), carbonyl groups (C=O), and carboxyl groups (COOH), respectively [35,36]. The assignment of the above functional groups is supported by the corresponding O1s spectra, which can be deconvoluted into several components at 531.5 eV and 533.0 eV. Although the assignment of functional groups by O1s spectra is not as straightforward as for C1s, these peaks can be attributed to O atoms in esters/carbonates, in ketons/ethers/alcohols, and in acids [37,38]. In addition, the O1s spectra for the NMO/rGO-M and NMO/rGO-U composite displays a signal at 530 eV, which is due to oxygen bonded to Ni/Mn in the ilmenite structure. All electrodes contain F element, which originates from the binding agent PVDF.

After the electrochemical test, the C1s and O1s spectra demonstrate the same signals as before the cycling. However, the signal intensities are subject to some changes, particularly those due to the functional groups. In addition, a new signal in the F1s spectra appears after the electrochemical testing. This signal located at 684.8 eV can be assigned to fluorine atoms in KF. This reveals that, after the electrochemical reaction, the surface of the electrodes is changed.

To quantify the changes in the surface composition of the electrodes, Table 1 lists the relative amount of the C, O, and F elements, as well as the functional groups. The comparison shows that the relative amounts of the functional groups are comparable for all the electrodes NMO/rGO-M, NMO/rGO-U, and rGO (Table 1). After the electrochemical test, the surface of rGO is enriched in carboxyl and carbonyl groups, while the hydroxyl/epoxide groups increase slightly. This means that the carboxyl and carbonyl groups are primarily involved in the electrochemical reaction, while the hydroxyl/epoxide groups are less reactive in the alkaline electrolyte. The different reactivity can be explained in terms of the interaction of the function groups with the electrolyte. In alkaline media, such as 6M KOH, the hydroxyl and carboxyl groups are unstable and they become deprotonated, resulting in the deposition of potassium hydroxide and carbonate salts [39,40,41]. This suggestion is supported by the amount of K element found on the surface using XPS (Table 1). Unlike hydroxyl and carboxyl groups, carbonyl groups are thought to be more likely to participate in pseudocapacitance reactions, storing and releasing electrons without ion exchange [42,43,44]. This means that in an alkaline electrolyte, carbon-rich surface film is formed on rGO during the electrochemical reaction. Given the strong decrease in the capacitance of rGO in the first cycles (Figure 5), it seems that the carbon-rich surface film grows predominantly up to 2000 cycles, an in the case when rGO was cycled at 240 mAg^−1^. After that, the stable cycling performance is achieved (Figure 5). Because of the sample depth analysed by XPS is up to 5 nm, the lack of the signal due to PVDF binder signifies that the film is thicker than 5 nm.

In comparison with rGO, the functional groups are slightly changed for the NMO/rGO-M and NMO/rGO-U composites. However, the amount of potassium from the electrolyte increases more, especially for NMO/rGO-M. This indicates that the composites readily adsorb potassium from the electrolyte, thus indicating better wettability of NMO/rGO-M electrodes. This is associated with the better electrochemical performance of NMO/rGO-M.

## 4. Conclusions

This study demonstrates the effect of mixing between rGO and ilmenite-type NiMnO_3_ on the energy/power density and long-term cycling stability of hybrid supercapacitors. The more homogeneous the mixture of rGO and NiMnO_3_, the better the capacitance, the energy and power density, and the long-term performance. This is achieved when rGO and NiMnO_3_ are mechanically mixed in a planetary centrifugal mixer. Contrary to the mechanical mixing, the ultrasonic treatment yields a non-homogeneous distribution with individual rGO particles and a large proportion of ilmenite particles covered with rGO. The contact between rGO and NMO is physical.

The NiMnO_3_/rGO composite obtained by mechanical mixing delivers an energy density of 15 Whkg^−1^ at a power of 1200 Wkg^−1^. When the composite is obtained by ultrasonic treatment, a lower energy density is achieved for the same power density. This is related to the surface and morphological changes in rGO that occur during the long-term supercapacitor cycling. In the composites, the rGO component is mainly attacked by the alkaline electrolyte solution, resulting in the enrichment of the rGO surface with carboxyl and carbonyl groups and the fracturing and wrinkling of the flat particles. The electrolyte-induced changes in the surface and morphology of the rGO component provoke the detachment of the rGO component from the NiMnO_3_ particles, leading to a loss of long-term cycling stability. The better properties of NMO/rGO-M than NMO/rGO-U reveal that the mechanical mixing in the planetary centrifugal mixer ensures a good contact between NiMnO_3_ and rGO particles, which is largely maintained after cycling.

In general, this study demonstrates that rGO has a more complex role in the capacitive performance of composites in alkaline media. The capacitive performance of rGO proceeds alongside with the surface reactions of carboxyl, hydroxyl, and carbonyl groups with KOH, leading to the wrinkling of the flat rGO particles. This directly influences the stability and efficiency of composite electrodes. Thus, the findings suggest that beyond electrical conductivity and initial mixing methods, maintaining a stable rGO-oxide interface during cycling is crucial for optimizing capacitive performance.

Furthermore, the enhanced performance observed in mechanically treated composites underscores a significant practical implication: costly procedures such as hydrothermal and ultrasonic treatments may not be necessary for effective composite formation. These insights could pave the way for optimizing composite materials and guide future advancements in scalable, cost-effective energy storage solutions.

## Figures and Tables

**Table 1:** Surface chemical compositions determined from XPS analysis for pristine electrodes and electrodes after 10,000 cycles.

Samples	C at.%	O at.%	K at.%	F at.%	C-O C-C	C=O C-C	O-C=O C-C
NMO/rGO-M	63.6	23.9	-	12.5	0.42	0.17	0.09
NMO/rGO-M (10,000 cycles)	55.9	31.7	8.7	3.7	0.35	0.20	0.10
NMO/rGO-U	64.9	22.3	-	12.8	0.38	0.14	0.09
NMO/rGO-U (10,000 cycles)	56.1	33.2	7.2	3.5	0.39	0.20	0.15
rGO	74.3	9.9	-	15.8	0.38	0.13	0.06
rGO (10,000 cycles)	53.1	34.8	6.7	5.4	0.46	0.25	0.19

## Data Availability

The original contributions presented in this study are included in the article/Supplementary Material. Further inquiries can be directed to the corresponding author.

## Supplementary Materials

The following supporting information can be downloaded at: https://www.mdpi.com/article/10.3390/ma18112460/s1, Figure S1: XRD patterns of (a) rGO; (b) NMO; (c) NMO/rGO-M; (d) NMO/rGO-U; Figure S2: Texture parameters of individual components (NMO and rGO) and composites NMO/rGO-M and NMO/rGO-U; Figure S3: CV plots of rGO at 1 mVs^−1^ and 50 mVs^−1^ before and after GCD test, Figure S4: Ex-situ XRD patterns of (a) NMO powder and electrodes after 10,000 cycles: (b) NMO/rGO-M, (c) NMO/rGO-U, and (d) rGO. The peaks due to the Ni foam and graphite are marked on the plot.; Table S1: Texture parameters of individual components (NMO and rGO) and composites NMO/rGO-M and NMO/rGO-U.
