# Supplementary material for: Mixing Approaches in Enhancing the Capacitive Performance of rGO-Based Hybrid Electrodes
Source: Materials (Basel). 2025 May 24;18(11):2460. doi: 10.3390/ma18112460 (PMC12156346; doi:10.3390/ma18112460)
Supplement: Supplementary file 1 [file materials-18-02460-s001.zip › materials-3623059-supplementary.pdf]

Supplementary Materials: Mixing Approaches in Enhancing the Capacitive Performance of rGO-Based Hybrid Electrodes

Svetlana Veleva[1], Delyana Marinova[2], Sonya Harizanova[2], Violeta Koleva[2], Elefteria Lefterova[1], Maria Shipochka[2], Ognian Dimitrov[1], Antonia Stoyanova[1], Radostina Stoyanova[2,*]

[1] Institute of Electrochemistry and Energy Systems, Bulgarian Academy of Sciences, 1113 Sofia, Bulgaria; svetlana_veleva@iees.bas.bg (Sv.V.); edl@iees.bas.bg (E.L.); ognian.dimitrov@iees.bas.bg (O.D.); antonia.stoyanova@iees.bas.bg (A.S.)

[2] Institute of General and Inorganic Chemistry, Bulgarian Academy of Sciences, 1113 Sofia, Bulgaria; manasieva@svr.igic.bas.bg (D.M.); sonya@svr.igic.bas.bg (S. H.); vkoleva@svr.igic.bas.bg (V. K.); shipochka@svr.igic.bas.bg (M.Sh.)

* Correspondence: radstoy@svr.igic.bas.bg

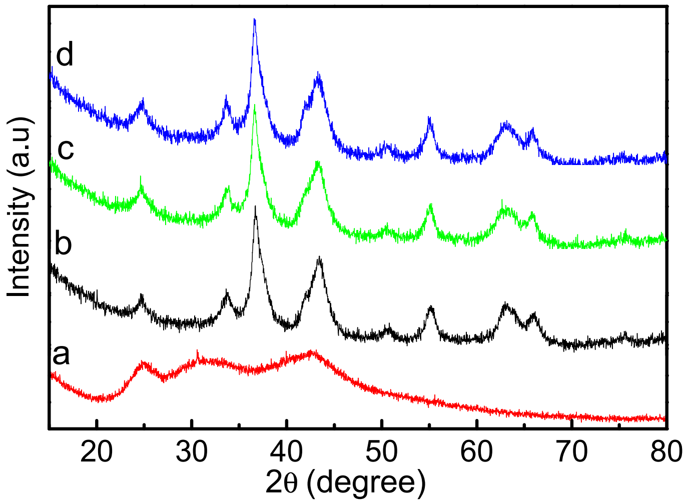

Figure S1. XRD patterns of: (a) rGO; (b) NMO; (c) NMO/rGO-M; (d) NMO/rGO-U

Table S1. Texture parameters of individual components (NMO and rGO) and composites NMO/rGO-M and NMO/rGO-U

**Samples**	**Specific surface area, m^2/g**	**Total pore volume, cm^3g^{-1}**
NMO/rGO-M	117	0.28
NMO/rGO-U	124	0.31
NMO [10]	128	0.35
rGO [28]	363	1.58

10. Soserov, L., Marinova, D., Koleva, V., Stoyanova, A., Stoyanova, R. Comparison of the properties of Ni–Mn hydroxides/oxides with Ni–Mn phosphates for the purpose of hybrid supercapacitors. *Batteries* **2022**, *8*, 51.
28. Harizanova, S., Tushev, T., Koleva, V., Stoyanova, R. Carbon-based composites with mixed phosphate-pyrophosphates with improved electrochemical performance at elevated temperature. *Materials*, **2023**, *16*, 6546.

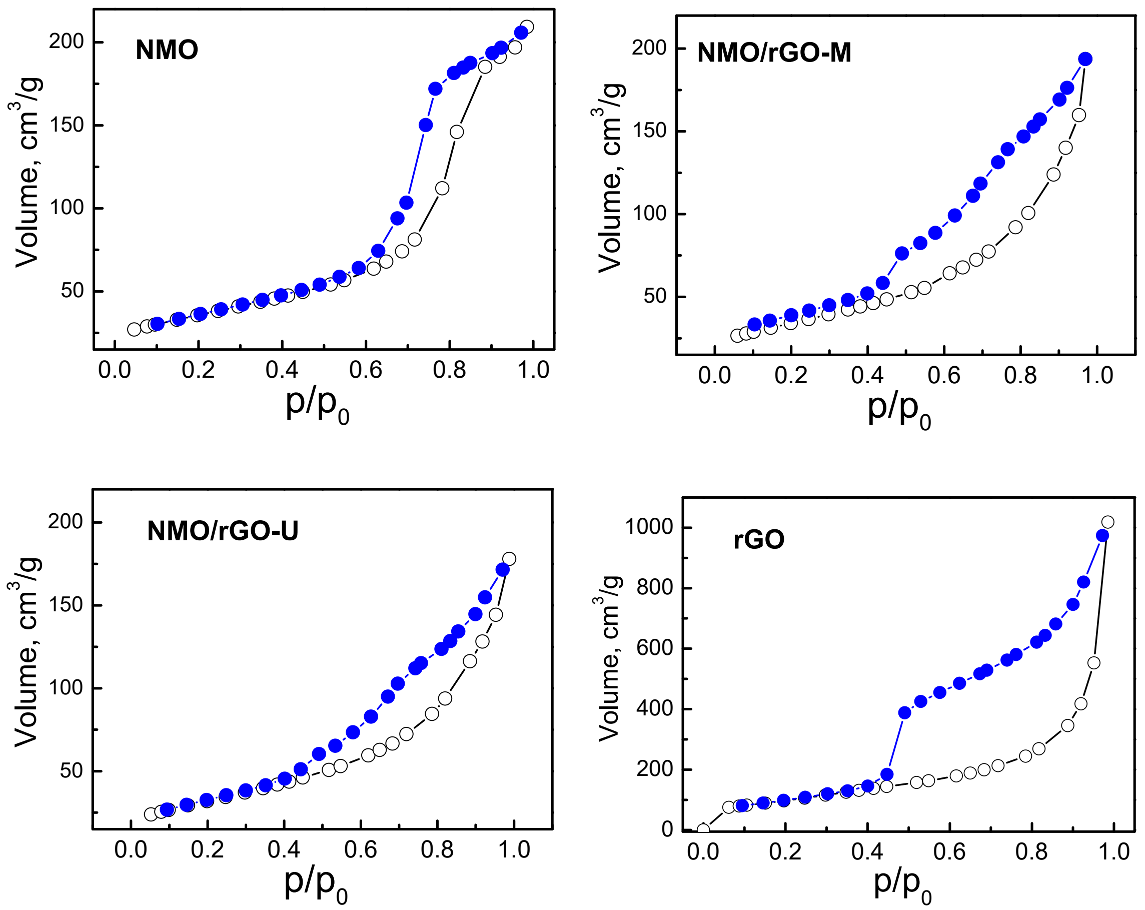

Figure S2. Nitrogen adsorption/desorption isotherms (open/full symbols) of NMO, NMO/rGO-M, NMO/rGO-U and rGO.

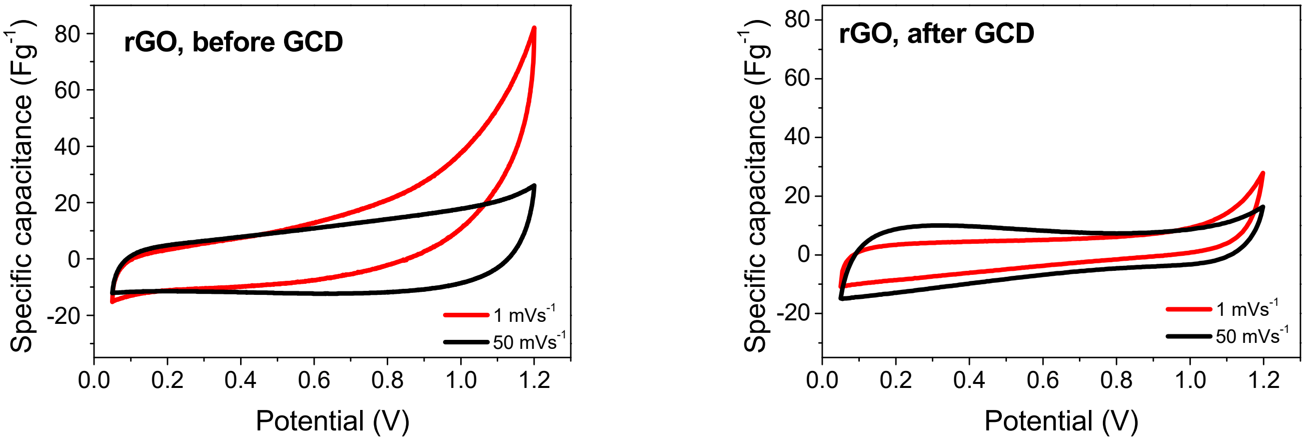

Figure S3. CV plots of rGO at 1 mVs^{-1} and 50 mVs^{-1} before and after GCD test.

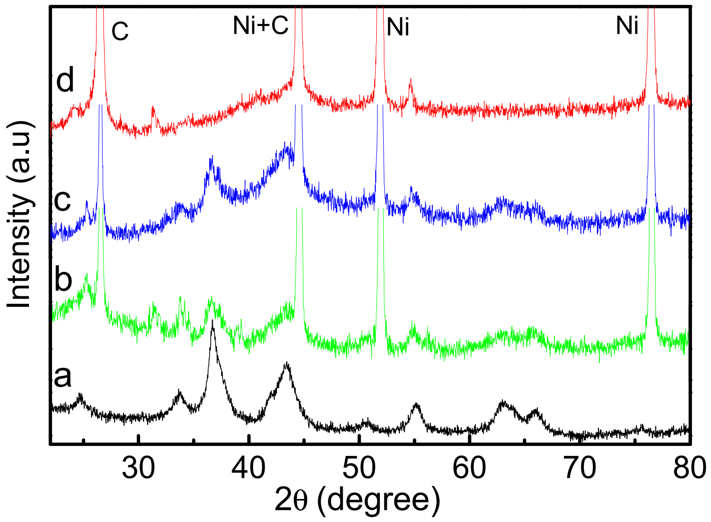

Figure S4. *Ex-situ* XRD patterns of (a) NMO powder and electrodes after 10000 cycles: (b) NMO/rGO-M; (c) NMO/rGO-U and (d) rGO. The peaks due to the Ni foam and graphite are marked on the plot.
